# An Integrated Micro- and Macroarchitectural Analysis of the *Drosophila* Brain by Computer-Assisted Serial Section Electron Microscopy

**DOI:** 10.1371/journal.pbio.1000502

**Published:** 2010-10-05

**Authors:** Albert Cardona, Stephan Saalfeld, Stephan Preibisch, Benjamin Schmid, Anchi Cheng, Jim Pulokas, Pavel Tomancak, Volker Hartenstein

**Affiliations:** 1 Institute of Neuroinformatics, ETH/University of Zürich, Zürich, Switzerland; 2 Max Plank Institute for Molecular Cell Biology and Genetics, Dresden, Germany; 3 Lehrstuhl für Genetik und Neurobiologie, University of Würzburg, Würzburg, Germany; 4 Automated Molecular Imaging Group, The Scripps Research Institute (TSRI), San Diego, California, United States of America; 5 Department of Molecular, Cell and Developmental Biology, University of California Los Angeles, Los Angeles, California, United States of America; 6 Janelia Farm Research Campus, Howard Hughes Medical Institute, Ashburn, Virginia, United States of America; University of Texas at Austin, United States of America

## Abstract

The analysis of microcircuitry (the connectivity at the level of individual neuronal processes and synapses), which is indispensable for our understanding of brain function, is based on serial transmission electron microscopy (TEM) or one of its modern variants. Due to technical limitations, most previous studies that used serial TEM recorded relatively small stacks of individual neurons. As a result, our knowledge of microcircuitry in any nervous system is very limited. We applied the software package TrakEM2 to reconstruct neuronal microcircuitry from TEM sections of a small brain, the early larval brain of *Drosophila melanogaster*. TrakEM2 enables us to embed the analysis of the TEM image volumes at the microcircuit level into a light microscopically derived neuro-anatomical framework, by registering confocal stacks containing sparsely labeled neural structures with the TEM image volume. We imaged two sets of serial TEM sections of the *Drosophila* first instar larval brain neuropile and one ventral nerve cord segment, and here report our first results pertaining to *Drosophila* brain microcircuitry. Terminal neurites fall into a small number of generic classes termed globular, varicose, axiform, and dendritiform. Globular and varicose neurites have large diameter segments that carry almost exclusively presynaptic sites. Dendritiform neurites are thin, highly branched processes that are almost exclusively postsynaptic. Due to the high branching density of dendritiform fibers and the fact that synapses are polyadic, neurites are highly interconnected even within small neuropile volumes. We describe the network motifs most frequently encountered in the *Drosophila* neuropile. Our study introduces an approach towards a comprehensive anatomical reconstruction of neuronal microcircuitry and delivers microcircuitry comparisons between vertebrate and insect neuropile.

## Abstract 2

A new software package allows for dense electron microscopy reconstructions of neuronal networks in the fruit fly brain, and reveals specific differences in microcircuits between insects and vertebrates.

## Author Summary

Brains contain a vast number of connections between neurons, termed synapses. The precise patterns of these synaptic contacts form the structural underpinning of electrical microcircuits responsible for animal behavior. Due to their small size, synaptic contacts can be conclusively shown using only high-resolution electron microscopy (EM). Therefore, complete series of ultrathin sections are required to reconstruct neuronal microcircuitry. The acquisition and analysis of EM sections (with 15,000 sections per millimeter of tissue) is practical only by computer-assisted means. In this article, we demonstrate the utility of the software package TrakEM2 to model interconnections of nerve fibers from consecutive EM sections and to efficiently reconstruct the neural networks encountered in different parts of a small brain, the early larval brain of the fruit fly Drosophila melanogaster. Neuronal networks are composed of patterns of axons and dendrites (neuronal extensions that transmit and receive signals, respectively), and using TrakEM2, we describe the most common motifs they form. Our study introduces an approach towards a comprehensive anatomical reconstruction of neuronal microcircuitry and delivers microcircuitry comparisons between vertebrate and insect brains.

## Introduction

The brain of all higher animals is formed by a large number of interconnected neurons. Typically, neurons are grouped into larger assemblies (“brain compartments”), such as brain stem nuclei or cortical layers in the vertebrate brain, or neural lineages in the insect brain [1],[2]. The analysis of the structure, development, and function of the brain can therefore proceed at two levels: the level of individual neurons and synapses, and the level of brain compartments. Compartments represent structural and functional modules; interconnected by bundles of axons, they form “macro-circuits” that control certain aspects of behavior. Unraveling macro-circuits has been the mainstay of classical vertebrate neuroanatomy and physiology. Present-day studies employing functional imaging (e.g., [3],[4]) walk in the foot steps of this approach, given that the signals registered by MRI or PET scanners (for these and all other abbreviations, see Table 1) reflect the activity of large numbers of contiguous cells [5].

The study of macrocircuitry informs us of how the brain is built, which “packets of information” may interact, where in the brain this interaction takes place, and what output channels are activated to elicit a behavior that is correlated with the observed macroscopic brain activity. Addressing macrocircuitry leaves the question of how nervous tissue operates in processing information unanswered. To tackle this problem, an approach is required that considers the structure and connectedness of the building blocks of the brain—i.e., the neurons, neurites, and synapses (“microcircuitry”). The way in which a given neuron is tuned to a specific input stimulus, or the pattern of activity triggered in this neuron when providing a specific input, depends on the distribution of excitatory and inhibitory synapses that connect the neuron with its neighbors [6]–[9]. Given the small size of neurites and synapses, the number and density of connections is immense. Calculations based on both light- and electron microscopic preparations point out that in mammalian brain, 1 mm^3^ contains more than 10^5^ neurons, more than 4 km of axon and 500 m of dendrite, and more than 700 million synapses [10]. The goal of the analysis of microcircuitry is to elucidate the geometric algorithms that describe the connectivity within small volumes of brains; based on these algorithms, one may hope we will be eventually able to model the neuronal activity and information flow pervading brain tissue while controlling sensory and motor activity of an organism.

Due to the small size of synapses and terminal neurites (0.1–0.5 µm), structural aspects of microcircuitry can be conclusively analyzed only electron microscopically. Traditionally, the acquisition and analysis of complete series of TEM sections required a considerable effort; as a consequence, studies of microcircuitry have mostly been restricted to small parts of neurons or neuropile compartments in (e.g., [11]–[14]). The problem is now becoming solvable, at least for small brains (or small volumes of large brains) with digital image recording and specialized software for both image acquisition and post-processing [15]–[18]. The resulting stacks of registered digital sections can be segmented and analyzed in their entirety. For the implementation of such a neuronal reconstruction pipeline, aimed at the analysis of microcircuitry of the *Drosophila* larval brain, we used the TrakEM2 open source software.

**Figure 1:**
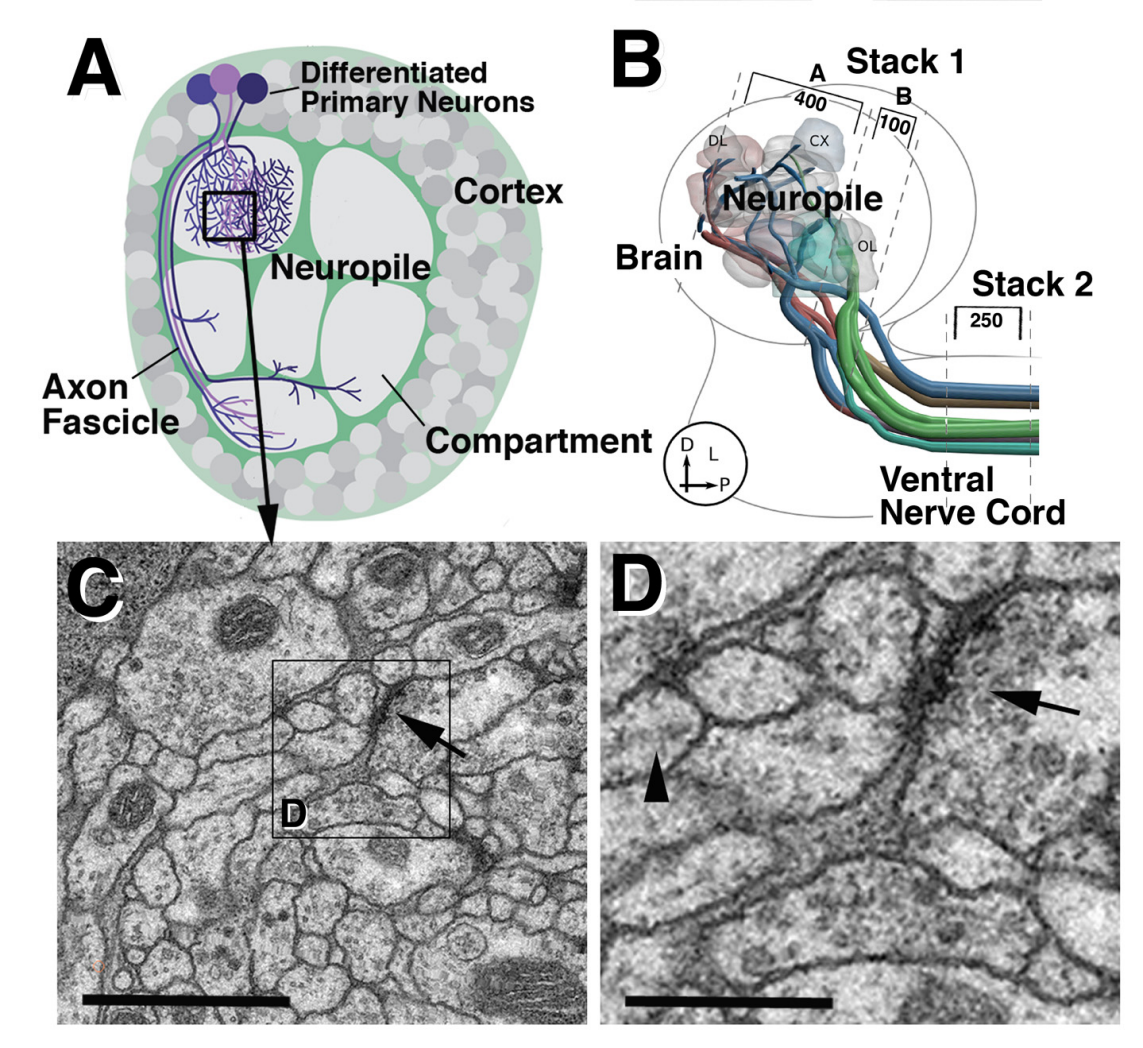
*Drosophila* first instar larval brain. (A) Schematic depiction of cross-section of one brain hemisphere, showing outer cortex of neuronal cell bodies in central neuropile, formed by neuronal processes (neurites). One lineage of primary neurons is highlighted in purple color. Processes of glial cells (green) surround the cortex and neuropile and form boundaries around compartments within the neuropile. (B) Schematic of larval brain and ventral nerve cord, indicating positioning and orientation of serial sections. The first stack contains the neuropile of one brain hemisphere in two closely adjacent sets of 400 and 100 sections, respectively. The second stack includes 250 sections of the ventral nerve cord (corresponding to approximately two consecutive neuromeres). Colored lines represent axon tracts connecting brain and ventral nerve cord (after 69). (C, D) Electron micrographs of sections of neuropile illustrating resolution that can be achieved at 5,000× primary magnification. Arrow in (C) points at synapse; arrow in (D) indicates presynaptic vesicles; arrowhead shows obliquely sectioned microtubule. At a resolution of 4 nm per pixel, these structures can be clearly resolved. Scale bars: 1 µm (C); 350 nm (D).

*Drosophila* serves as a favorable model system in which molecular pathways involved in a wide range of events, from neural stem cell proliferation, cell fate determination, neurite pathfinding, and neurite connectivity, can be studied (e.g.,[19]–[22]). With the help of sophisticated transgenic constructs (e.g., Gal4 lines; [23]), one can target specific cell types, labeling these cells, or manipulating them genetically. As a result, *Drosophila* also represents a great model system to study systems-level questions, such as how neural circuits develop or control behavior. Finally, *Drosophila* (and insects in general) offers the advantage that its nervous system is formed by a relatively small number of genetically and structurally defined modules, the neural lineages [1],[24]–[25]. Early in development, a set of dedicated neural progenitors, called neuroblasts, segregate from the ectoderm and subsequently proliferate to form the neurons and glial cells of the CNS. Each neuroblast produces an invariant set (“lineage”) of neurons; these neurons form a genetic as well as a structural “module” of the brain (Figure 1). Thus, axons of neurons belonging to the same lineage form a cohesive tract and arborize within discrete compartments of the brain. The axon tracts formed by neural lineages, and recognized from early larval stages into the adult brain, represent the structural/developmental units of brain macrocircuitry.

Lineage tracts represent an invariant, easily recognizable system of structural landmarks onto which smaller elements—i.e., microcircuits or individual neurons—can be mapped. We argue that for the efficient analysis of microcircuit data, it is advantageous to follow an approach that integrates light microscopic landmark structures, such as lineage tracts (the “macrocircuitry”), with the analysis of TEM datasets. The reconstruction of microcircuitry from serial TEM material involves two main operations. The first is to segment the profiles of neurites through many consecutive sections to establish branch points and synaptic contacts. Given the amount of time involved in manual segmentation (or proofreading of automatically segmented material; [18]), there are currently two different strategies of serial TEM data analysis. One (“sparse strategy”) is to focus on a defined, relatively small set of neurons, such as sensory afferents, or motor neurons, and trace their profiles and synaptic outputs/inputs in their entirety. The other strategy, introduced in this article, is to break down the overall TEM stack volume into smaller “microvolumes,” in the range of 2×2×1.5 µm up to 5×5×5 µm, and reconstruct all profiles contained within these microvolumes (“dense strategy”). From these small volumes, structural parameters of terminal neurite connectivity (e.g., diameter, orientation, and density of terminal neurites; structure, size, and density of synapses) can be extracted. Irrespective of the strategy (sparse versus dense segmentation) chosen, an additional step is to put the connectivity data resulting from the segmentation into the context of brain macrocircuitry: what brain compartment is the microcircuit located in, what are its input and output channels, how does it compare to microcircuits located at other positions in the brain. To be able to make these connections, the operator has to be able to repeatedly switch between the EM and LM level and to rapidly navigate from one location/compartment to another.

The TrakEM2 software used in this study combines all the components required for data acquisition, registration, navigation, and segmentation ([26],[27]; http://www.ini.uzh.ch/~acardona/trakem2. html). TrakEM2 enables the analysis of a brain volume at the microcircuit level (synapses, individual neurite branches) along with the macrocircuit level (brain compartments, axon fascicles). We illustrate the use of TrakEM2 towards this end by applying it to the brain of the first instar (L1) larval *Drosophila* brain. As raw data we use two stacks of stitched and registered EM sections, one containing 500 sections that include most of the neuropile of one brain hemisphere, and another one of 250 sections including one complete abdominal neuromere of the ventral nerve cord. We have cropped out several small volumes and evaluated the structure and connectivity of the terminal neurites contained within these volumes. Furthermore, we have registered a confocal stack with the brain TEM stack and evaluated the accuracy with which we can translate light microscopic features from the confocal stack to the TEM stack. Our study is intended to develop an approach towards a comprehensive anatomical reconstruction of neuronal microcircuitry.

## Materials and Methods

### EM Histology

Freshly dissected first instar fly brains were fixed in 4% paraformaldehyde and 2.5% glutaraldehyde in 0.1 M freshly made phosphate buffer (PB; ph 7.3) for 24 h at 4°C. Brains were then rinsed 5×10 min in 0.1 M PB, postfixed in 1% osmium tetraoxyde in 0.2 M PB for 60 min at 4°C, and rinsed 4×10 min in distilled water. Dehydration was done through an acetone series (10 min 50%, 10 min 70%, 10 min 96%, 3×10 min 100%). Preparations were embedded in Epon resin at room temperature as follows: 2 h in 1∶3 Epon:acetone, 3 h 2∶2, overnight 3∶1, overnight pure Epon. Blocks were cured for 16 h at 60°C. 60 nm serial sections were cut on a Leica UC8 ultratome and collected in ribbons onto pioloform-coated single slot copper grids. Grids were contrasted in 8% uranyl acetate (30 min at 60°C) and in Reynold's lead citrate [28].

### Immunohistochemistry and Confocal Imaging

Larval brains of a line in which the neurons expressing pigment dispersing factor (PDF) were labeled by a Gal4-driven GFP reporter were dissected in PBS and fixed in PBT (PBS with 0.1% Triton X-100) containing 4% formaldehyde for 30 min at room temperature. A mouse anti-Neurotactin antibody ([29]; 1∶10; Hybridoma Bank) was used to label neurons and axon tracts. Secondary antibodies were purchased from Jackson Laboratory and used at the manufacturer's recommended concentrations. Stained brains were mounted in Vectashield (Vector Laboratory; H-1000). Confocal images were taken on a Biorad MRC1024ES microscope using Laser sharp version 3.2 software. Complete series of optical sections were taken with a 40× oil immersion lens at 1 µm intervals. Images were analyzed using the ImageJ software.

### Image Acquisition

The imaging of hundreds of sections at sufficient resolution to resolve details like synapses used to pose a major problem in high-throughput transmission electron microscopy (TEM). The software package Leginon [30] automates TEM image acquisition of multiple sections and large tissue areas. We used an FEI electron microscope equipped with a Tietz camera and a goniometer-powered mobile grid stage. Leginon software automatically controls every component of the electron microscope and attached camera. The acquisition starts by inserting a grid with about 10 serial sections into the microscope. Leginon images the entire slot of the grid at low resolution and offers a grid atlas for manually or semi-automatically picking the tissue areas of interest in all sections. Then Leginon automatically adjusts the stage, power, magnification, and camera to acquire the necessary sets of high-resolution image tiles that cover all areas of interest. For our larval neuropile sections, we chose a magnification of 5,600× binned at 2, delivering a resolution of 4 nm/pixel.

### Image Volume Composition: Montaging Image Tiles Within and Across Serial Sections

Acquired image tiles carry associated stage position coordinates, but these alone would result in suboptimal montages. First, we correct for lens deformations, which are constant to all tiles. For the purpose, we extract scale-invariant-feature-transform (SIFT) features [31] from nine heavily overlapping images and then estimate and apply the correcting transformation to all tiles [32]. The correction of lens deformation greatly facilitates the montaging of image tiles. Each section presents independent non-linear deformations, generated during sectioning and also induced by heat while imaging. No ground-truth is available for the correction of these deformations. Our observations indicate that, generally, section-wide gross deformations in consecutive sections are mostly independent and that local non-linear deformations contain translations smaller than the dimensions of the image tiles. An approach that registers image tiles all-to-all within and across sections would, to a considerable extent, cancel out all independent deformations. We approximate this ideal registration with an as-rigid-as-possible tile-wise registration method. We extract SIFT features from all image tiles and search for correspondences with their neighboring tiles within the section and with tiles in the previous and subsequent sections in the series. We estimate a rigid transformation model for each tile, simultaneously from all tile-to-tile feature correspondences. An iterative optimizer relaxes all tile-to-tile correspondences until the sum of their square inter-distances is minimal. From this configuration, we estimate a non-linear transformation for each individual section using the Moving Least Squared method [33] with tile centers as control points. With our approach, imaged objects (such as sectioned neural arbors) that span over multiple tiles within one tissue section are as smoothly continuous over tile boundaries as possible, while preserving maximum continuity across sections with minimal deformation applied to each individual image [27].

### Data Storage, Retrieval, and Navigation

The storage, retrieval, and navigation of a terabyte-sized dataset of TEM images by a human operator in real time is a difficult and time-consuming task, even when considering small objects like the *Drosophila* L1 brain. We have found a successful approach in the combination of two main factors: (1) the means to browse through large areas and volumes at sufficiently high speed, and (2) the ability to zoom in and out at very high speed, for the purpose of obtaining a positional reference. We approach browsing with mipmaps, that is, precomputed multiresolution images, which match the current view magnification and are thus optimal for data transfer and display [28],[34].

### Data Analysis: Segmentation Tools

Our immediate goal in analyzing the TEM stack was 2-fold: on the one hand, we want to crop out small volumes, in the range of 5 µm across, for which we manually dense-segment every process and synapse. Secondly, we wanted to identify and segment larger structures, such as axon bundles and compartment boundaries, which together form a framework of landmarks to which the microvolumes could be related. To efficiently segment these different structures by hand, we employed a small set of segmentation data types: an “area list” (a list of 2D multi-area objects, one per section, can represent individual neurites but also large objects like compartments), a “ball” (a list of x,y points with a radius, sitting on any section, can represent a specific landmark, like point of intersection of tracts), a “pipe” (a serial sequence of points, each point with an x,y coordinate on a specific section, and with a radius that defines a tube passing through all points, can represent axon tract), and a series of other convenience objects like floating text labels (with associated x,y coordinates and on a specific section).

### Data Analysis: Object Hierarchy

Densely segmented volumes, even at the scale of microvolumes, contain a large number of separate objects that stand in specific relationships to each other. We call the objects of a given brain the “object hierarchy” of this brain. The structural elements of the object hierarchy are user-provided; the L1 brain, for example, contains, among others, the high-order elements “neuropile compartments,” “neuropile tracts,” “lineages,” and “neurons.” High-order elements include further, lower tiers of elements; a neuron, for example, forms primary branches A, B; these in turn have secondary branches A1, A2, … and B1, B2 … etc. Each branch forms a set of presynaptic and/or postsynaptic sites, through which a given branch of a given neuron communicates with a certain branch of another neuron. In other words, the effective manipulation of the multitude of objects included in a segmented brain (microvolume) demands a strategy that groups these objects into recursively smaller and smaller groups, effectively collapsing the complexity to an arbitrary level at which high-order elements (like “neurons” or “branches”) become the elements to operate on: to measure, to hide/show, to visualize in 3D, to color, to highlight, etc. The result is a hierarchal tree of abstract objects: a “neuron” is represented as a composition of lower-level objects like “soma” and “arbor,” each of which in turn is composed of further lower-level objects like “nucleus,” “cytoplasm,” “axon,” “dendrite,” and so on, until reaching the level of primitive object instances (whose terminology follows a controlled vocabulary, such as “area list” or “pipe”). An “area list,” for example, would represent the series of 2D sections of a nucleus or synapse, which has been created by manually or semi-automatically painting with mouse movements over TEM images.

The object hierarchy window is set up in a manner that will ultimately allow one to export the data in “sif” format into programs designed to perform network analysis. Thus, tools like “Neuron” (http://www.neuron.yale.edu/neuron/) are designed to accept data sets composed of large numbers of individual elements connected via defined synapses and compute neural circuit simulations using Hodgkin-Huxley models (integrate-and-fire models).

### TrakEM2 Usage for Neuronal Reconstruction

TrakEM2 simultaneously presents two ways of browsing and manipulating the data: on the one hand, the tree hierarchy of abstract groupings, with the actual segmentation object instances at the tips of the tree (“object hierarchy window”), and on the other hand, a 2D display (“canvas”) for the manipulation of the data of the segmentation objects (to fill in an area, to point-and-click to add spheres, or lines, etc.) The 2D canvas is a view of the list of serial sections, which can be any series of images such as image stacks from confocal microscopy or registered histological or TEM serial sections. Via the 2D display, the images themselves are manipulated. The key operations for serial section TEM are batch-importing, image stitching within a section, registration of adjacent sections, and contrast adjustment. Our image registration approach preserves as much as possible the dimensions of each individual image, avoiding the introduction of artifactual image deformations [27]. Both image volumes and segmented objects may be visualized in 3D for spatial analysis, using the ImageJ “3D Viewer” [35].

### Importing Extrinsic Objects

One of the requirements for an efficient serial section TEM analysis is the recognition of structures that have already been characterized at the light microscopic level. These structures provide the context for the analysis of microstructures like synapses and statistics of arborizations, etc. Currently, no algorithms exist for generic automatic cross-modal image registration; that is, no unsupervised algorithm can recognize structures both at the TEM and at confocal images. TrakEM2 offers a simple 3D nonlinear image registration approach based on user-picked fiduciary marks, common between TEM and confocal images. From the fiduciary marks, a nonlinear transformation is estimated using the Moving Least Squares method [33] for 3D affine transformation. The confocal image stack is thus brought into register with the TEM sections. Then a re-sliced 50 nm section of the confocal image stack is overlaid on top of the TEM section, using color composites.

## Results

### An Integrated EM and LM Approach to the Analysis of Brain Circuitry

We prepared an uninterrupted series of TEM sections of a small brain, the *Drosophila* first instar (L1) brain, recorded digital images of a complete brain hemisphere at a high enough resolution to reconstruct synapses and fine processes, and registered these images so they can be navigated like a confocal stack. The *Drosophila* L1 brain hemisphere has a diameter of approximately 50 µm. It consists of an outer cortex of neuronal cell bodies and a central neuropile containing the branched neurites and synapses (Figure 1A). The diameter of the neuropile measures 30 µm. In a first series of TEM sections we focused on the neuropile; 500 sections of 60 nm thickness included the entire neuropile of one brain hemisphere. In addition, we sectioned transverse slice of the ventral nerve cord, containing 250 sections. For image capturing, we used the software Leginon (Automated Molecular Imaging group at the Scripps Institute, San Diego, CA). To clearly resolve ultrastructural details such as neurotubules (about 25 nm diameter) and synaptic vesicles (30–40 nm diameter), we aimed at a resolution of approximately 3–4 nm per pixel, which can be achieved at a magnification of 3,000–5,000× (Figure 1). At that resolution, the complete data stack including the neuropile of one brain hemisphere amounts to approximately 1 terabyte; for the entire L1 brain, the size would be approximately 5 terabyte.

**Figure 2:**
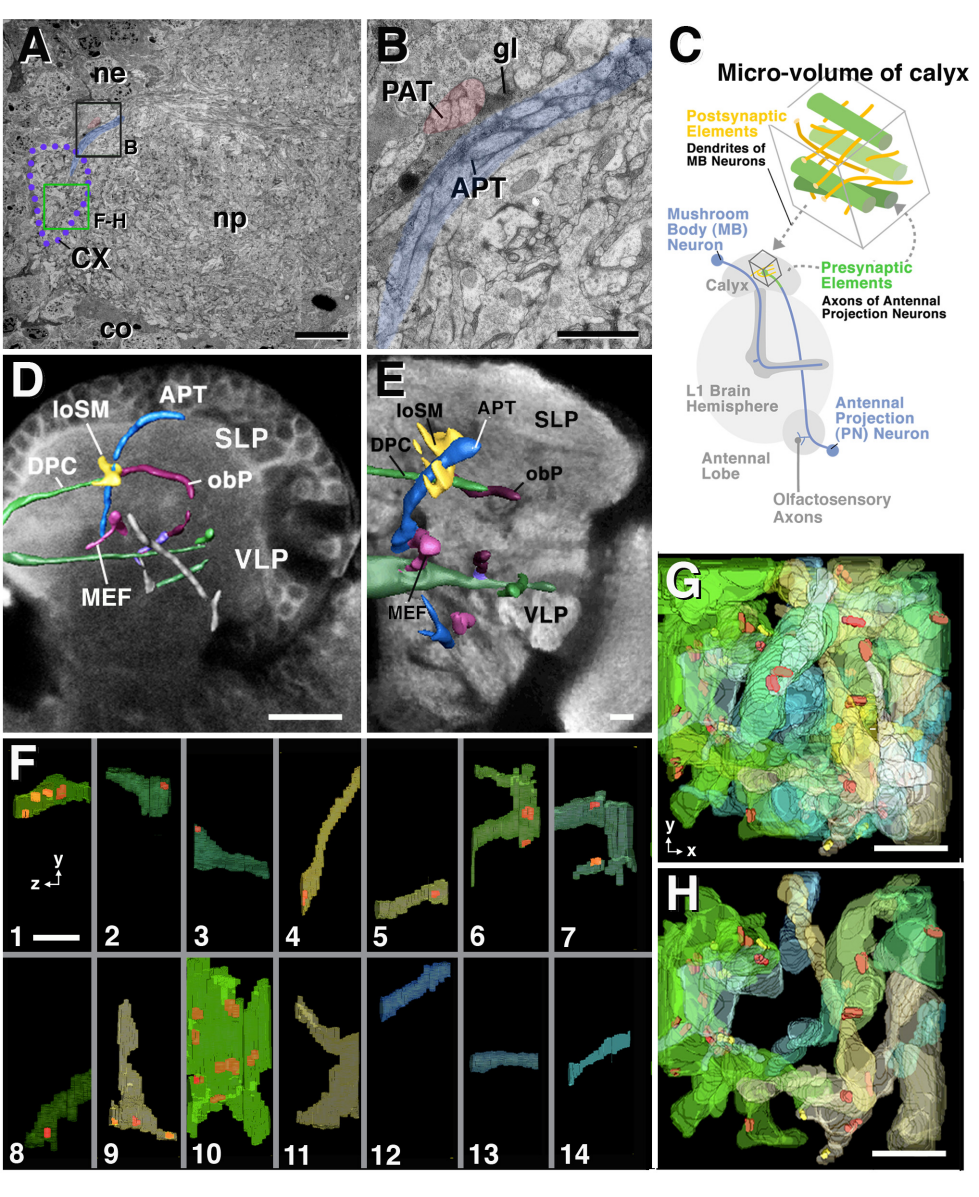
Microvolumes and neuropile landmarks. (A, B) Montage of electron micrographs of representative brain section (A; co, cortex; ne, neuronal cell body; np, neuropile). Note that this and all other electron microscopic images of the figures presented in this article are a composite of multiple tiled digital photographs stitched together with the TrakEM2 software, as explained in Materials and Methods. Occasional slight discontinuities in image brightness (as for example at lower right corner of panel A) correspond to borders of tiles. Bundles of long axons can be recognized and identified with specific, lineage-related fascicles known from confocal microscopy. Upper boxed area, shown at higher magnification in panel B, contains branch of antenno-cerebral tract (APT; blue) and adjacent axon tract formed by one of the DPM lineages (PAT). Note glial processes (gl) accompanying axon fascicles. Green box in (A) demarcates size and position of a microvolume that was cropped out of stack for further analysis (see panels F–H). Dotted purple line demarcates calyx (CX) compartment. (C) Schematic drawing showing L1 brain hemisphere with circuit consisting of antennal lobe-calyx-spur/lobes of mushroom body. Fascicles forming this circuit (e.g., APT, mushroom body) can be identified in TEM stack and help to interpret short segments of neurites contained within microvolumes (e.g., microvolume from within the calyx). (D, E) Confocal cross-sections of first instar larval brain hemisphere (D) and adult brain hemisphere (E) labeled with anti-Brp to visualize neuropile. Set of lineage-related axon fascicles that can be followed from first instar to adult are rendered in different colors. These fascicles (among them the APT shown in panels A, B) form a system of invariant landmarks that can be recognized in confocal stacks and TEM stacks. Abbreviations: APT, antenno-protocerebral tract; DPC, dorso-posterior commissure; loSM, longitudinal superior-medial fascicle; MEF, medial equatorial fascicle; obP, oblique posterior fascicle; SLP, superior lateral protocerebrum; VLP, ventro-lateral protocerebrum. (F–H) 3D views of selected objects within the calyx microvolume. Microvolume contained 42 large diameter (>0.2 µm) neurite segments. Of these, 14 were randomly picked and are shown individually in panels F1–F14 (volume in side view; y and z indicate direction of axes of volume). (G, H) Top view of large diameter neurite segments combined (G: all 42 segments; H: 14 segments shown individually in panel F). Red dots indicate position of presynaptic sites. Scale bars: 4 µm (A); 0.5 µm (B); 10 µm (C, D); 1 µm (F, G, H).

Our strategy of “dense neuropile analysis” was to extract from the TEM image volume multiple smaller “microvolumes,” in the range of 2×2×1.5 µm up to 5×5×5 µm (Figure 2A), and reconstruct all profiles contained within these microvolumes. TrakEM2 allows us to efficiently crop out such microvolumes and re-register them automatically. Contained within a microvolume are the contiguous short segments and terminal branches of many neurons and their synaptic contacts (Figure 2F–H; Figures S1, S2). As shown below, the dense analysis of these objects can shed light on a number of fundamental parameters of microcircuitry. Furthermore, we argue that the reconstruction and analysis of objects from stacked TEM images should be guided by known “macroarchitectural” brain landmark structures. In other words, the interpretation of the pattern of neurites contained within a given microvolume will be made easier by establishing position, input, and output relationship of that volume (Figure 2C). TrakEM2 embeds the analysis of the TEM stack at the microcircuit level into a light microscopically derived three-dimensional framework of landmark structures. This framework is provided by the invariant pattern of compartment boundaries and lineage-related axon tracts, many of which have been identified in brains of all developmental stages ([36]–[40]; Figure 2D, E). Lineage related primary axon tracts (PATs) in a L1 brain contain 5–20 tightly bundled, straight axons that originate and terminate at defined positions. In many cases, PATs of several contiguous lineages converge and form large fascicles, such as the antenno-cerebral tract, peduncle, medial and lateral equatorial fascicle, or posterior-lateral fascicle. Fascicles are all associated with high concentrations of glial processes, which makes it easier to identify them in the TEM stack (Figure 2B). Glial densities also accompany many of the compartment boundaries [41]. Fascicles and segment boundaries (“macrolandmarks”) are segmented and become the objects of an “intrinsic macro-model” of the brain contained within the TEM stack.

**Figure 3:**
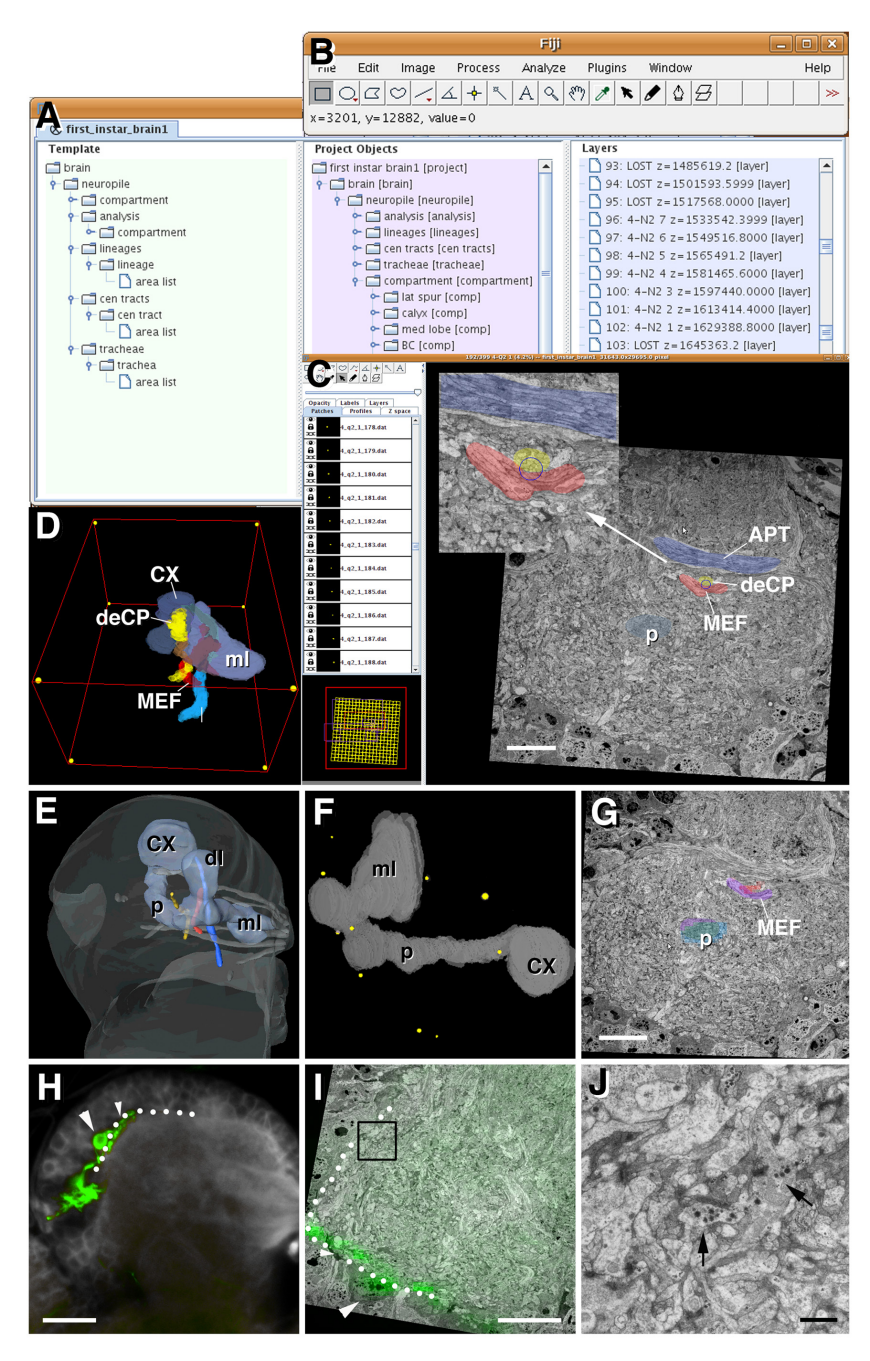
The graphical user interface of TrakEM2. (A) The object hierarchy window displays segmentation data types (left column, green), all segmented objects captured with these types (center column, magenta), and all layers along the *z*-axis (right column, blue). (B) ImageJ tool box. (C) The 2D canvas presents the TEM (or any other) raw data stack. The user can scroll and navigate through all sections displayed in this window. The yellow grid at bottom left of panel shows all of the image tiles that constitute the montage of the section shown. When zoomed in, a red frame indicates the area shown on canvas; by dragging the red frame, the user can navigate to the desired parts of the montage. Segmentation and annotation of identified structures (e.g., compartments and lineage tracts) is performed in the 2D canvas window. Shown are outlines of four identified axon fascicle (APT, antenno-protocerebral tract, blue; deCP, descending central protocerebral fascicle, yellow; MEF, medial equatorial fascicle, red; p, peduncle of mushroom body, light blue). Points of intersections of fascicles, such as the one between deCP and MEF (blue circle), serve as fiduciary marks with which different stacks are registered. (D) The interactive 3D viewer windows show selected objects segmented from the TEM stack. Shown as examples are the same elements depicted as sections in panel C [mushroom body (CX, calyx; ml, medial lobe), MEF fascicle, deCP fascicle]. (E–J) Registration of confocal stack to TEM stack. (E) 3D digital model of larval brain hemisphere derived from confocal stack. Shown are brain surface (light gray), mushroom body (light blue), and axon fascicles (dark gray). Set of fascicles highlighted in TEM stack (see panels C, D) are shown in corresponding colors. (F) 3D digital model of mushroom body, showing location of fiduciary marks (yellow) identified in both TEM stack and confocal stack. (G) Representative section of TEM stack. Shaded in purple are profiles of two axon fascicles (p, peduncle; MEF, medial equatorial fascicle) as they appear in the TEM section. Superimposed in red and blue are profiles of corresponding fascicles from confocal stack that was registered with TEM stack (using fiduciary marks shown in F) and digitally re-sliced along the plane of sectioning of the TEM stack. Note high degree of overlap between TEM profiles and “imported” confocal profiles. (H) Representative section of confocal stack of one brain hemisphere. Shown in green are GFP-labeled PDF neurons. The cell bodies of these neurons are located in the dorso-lateral cortex; axons form bundle that extends along the dorso-lateral cortex-neuropile boundary (white dotted line). Large arrowhead points at one cell body, small arrowhead at axon. (I) Section of registered, re-sliced confocal stack projected upon corresponding EM section. Cell body and axon are indicated by large and small arrowhead, respectively; boundary between dorso-lateral cortex and neuropile is shown as white dotted line. (J) Magnified view of area boxed in (I). Arrows point at profiles of neurites containing (neuro-secretory) dense-core vesicles. Based on their proximity to the position of PDF neurons, it is probable that these profiles correspond to branches of PDF neurites. Scale bars: 5 µm (C, G, I); 10 µm (H); 1 µm (J).

TrakEM2 allowed us to seamlessly transition from the light-microscopic (“macrocircuitry”) to ultrastructural level (“microcircuitry”) of brain analysis. The graphic user interface of TrakEM2 features three simultaneously active windows: the object hierarchy window, the 2D (raw data) canvas, and the 3D viewer (Figure 3). The digitized TEM stack of the larval brain is opened as a “project” in TrakEM2 and appears in the 2D canvas, where it can be navigated in the manner of a three-dimensional map [26]. Landmark structures that are segmented within the TEM stack are represented as nodes of a hierarchical tree in the object hierarchy window (Figure 3A). From within the object hierarchy window, individual objects can be activated or inactivated, and properties of their digital rendering, such as color or transparency, can be changed. An object (or any number of objects) activated from within in the object hierarchy window will appear as an outline overlaid upon the electron microscopic images visible in the 2D canvas window (Figure 3C). At the same time, a surface rendered 3D digital model of the object can be displayed in the 3D viewer (Figure 3D). Alternatively, an object can be activated from within the 2D canvas and is then identified in the object hierarchy window. As a result, we were able to focus at the ultrastructural level on any part of the neuropile in the 2D canvas and at the same time get orienting cues from the intrinsic macro-model (displayed in the 3D viewer) about the exact position relative to compartment boundaries and axon fascicles.

TrakEM2 also offers the possibility of adding additional macrostructures imaged from other (“extrinsic”) larval brains. Thus, a confocal stack of a L1 brain in which a certain brain object (e.g., cell type, or lineage), aside from the landmark structures, is labeled by antibody or reporter construct is opened as an additional, separate image volume. The landmark structures define a set of fiduciary marks that allow one to register the extrinsic confocal stack with the TEM stack. Following the registration, all structures that are labeled, or manually segmented as objects, in the extrinsic confocal project are overlaid with the TEM project. In the example shown in Figure 3E–J, the confocal stack of a brain in which the four neurons expressing the peptide PDF (pigment dispersing factor) were labeled was merged with the TEM stack. We used the points of intersection of 14 axon fascicles, with each other or compartment boundaries, as fiduciary marks for registering the confocal stack with the TEM stack (Figure 3E, F). To assess the accuracy of the registration, we first determined how closely the position of corresponding axon fascicles segmented from the TEM stack and the confocal stack matched. Shown in Figure 3G are the profiles of the MEF (medial equatorial fascicle) and dePF (descending protocerebral fascicle) as they appear in the TEM stack (outlined in magenta). Overlaid in blue (peduncle) and red (MEF) are the profiles of the corresponding fascicles that were segmented in the confocal stack and, following registration, overlaid upon the TEM stack. In most sections, as the one shown in Figure 3G, the profiles (which measure less than 2 µm in diameter) overlap. Profiles of the mushroom body peduncle (diameter: 5 µm) segmented from the confocal section overlap more than 50% with the peduncle derived from the TEM stack throughout all sections. The accuracy of registration can be further improved by refining and adding more fiduciary marks. Of course, one cannot expect that, employing the approach introduced here, a single neurite (whose diameter averages around 0.25 µm) segmented from a confocal stack will “fall” precisely over the corresponding counterpart within the TEM stack. For that not to happen, variability between brains (at the level of individual neurons) in itself is reason enough. However, the import of labeled objects from confocal stacks will assist significantly in identifying specific neurons, as shown in the following for a small group of neurons, the PDF neurons.

A GFP reporter expressed under the control of the PDF gene promoter [42] labels four neurons located in the dorso-lateral L1 brain hemisphere. Figure 3H and 3I illustrate the PDF neurons as they appear in a confocal section (3H) and overlaid upon the EM stack (3I). The main axons of the four neurons converge and fasciculate, extending as a thin transverse bundle along the dorso-lateral neuropile boundary. Dendritic branches of the PDF neurons branch off towards the larval optic neuropile (outside the plane of section); numerous short secondary and tertiary axonal branches are formed along the length of the PDF bundle into the CPLd compartment. PDF neurons belong to the class of peptidergic neurons, all of which are characterized ultrastructurally by prominent dense core vesicles filling the entire neurite tree. Peptidergic neurites innervate the neuropile quite sparsely; the overall number of peptidergic neurons in the L1 brain is in the order of 50 per hemisphere (reviewed in [43]), out of 1,500 neurons. Therefore, the profiles with dense core vesicles (Figure 3J) that we identify in the TEM stack in the domain occupied by the overlaid profiles of PDF neurons imported from the confocal stack most likely belong to the PDF neurons inherent to the TEM stack.

### *Drosophila* Brain Microarchitecture: Generic Classes of Neurites

**Figure 4:**
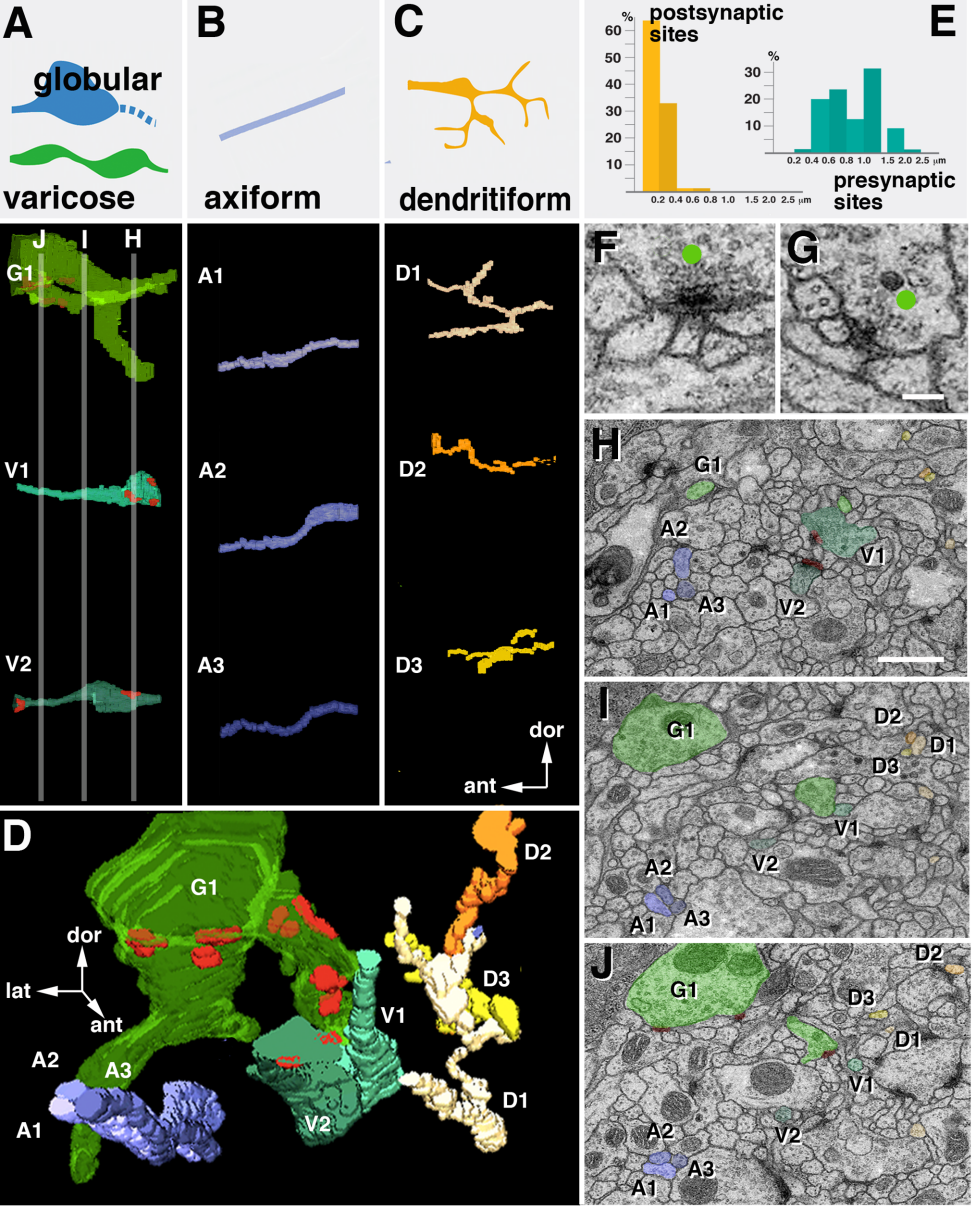
*Drosophila* neuropile ultrastructure. (A–C) Generic types of neurites (globular/varicose, axiform, dendritiform) encountered in all regions of the neuropile. Upper panels of columns A, B, C show schematic representations of these neurites; lower panels show 3D digital models of representative neurite segments for corresponding classes, segmented from VNC microvolume (G1, globular neurite; V1, V2, varicose neurites; A1-3, axiform neurites; D1-3, dendritiform neurites). Elements are shown in lateral view; arrows indicate orientation (ant, anterior; dor, dorsal) relative to body axes. Red dots indicate position of presynaptic sites. (D) 3D digital model of all neurite segments shown in (A–C) as they are situated in VNC microvolume. Volume is shown in dorso-posterior view; arrows indicate axes (ant, anterior; dor, dorsal; lat, lateral). (E) Correlation between frequency of presynaptic and postsynaptic sites and neurite diameter. Presynaptic sites (blue; right) are predominantly found on large diameter profiles, which correspond to thick segments of varicose and globular neurites. Postsynaptic profiles (yellow, left) almost exclusively belong to thin dendritiform neurites. (F, G) Sections of two typical polyadic synapses. Green dots indicate presynaptic profile, characterized by T-bar (synaptic ribbon) and synaptic vesicles. Presynapses are contacted by multiple, thin branches of dendritiform processes. (H–J) Representative EM sections of VNC microvolume at three different levels along the antero-posterior-axis; levels are indicated by gray lines in panel A. Profiles of neurites shown in (A–D) are shaded in the corresponding colors and identified by the corresponding annotations (G1, V1-2, A1-3, D1-3). Scale bars: 0.1 µm (F, G); 1 µm (H–J).

We have segmented and reconstructed neurites in five microvolumes located in different compartments (calyx and spur of mushroom body, dorso-lateral protocerebrum, and dorso-lateral domain of ventral nerve cord). As outlined above, the dense reconstruction of microvolumes yields information regarding structural parameters such as neurite diameters, directionality, branching, and synapse placement. Our data show that brain ultrastructure is conserved in several aspects in all regions sampled. We were able to identify four classes of neurites in each of the microvolumes (Figure 4). The first class, termed “axiform neurites,” is comprised of straight, unbranched processes of even diameter, ranging between 0.2 and 0.4 µm (Figure 4B, D). Axiform neurites form bundles originating in the cortex; they correspond to the primary axon tracts (PATs) emitted by the neurons belonging to one lineage. Within the neuropile, groups of PATs typically coalesce to form larger assemblies (fascicles; see examples of MEF or APT in Figure 3C). The second class of neurites (“varicose neurites”) consists of branched processes that alternately decrease and increase in diameter along their trajectory (Figure 4A, D). Thick segments (swellings or “varicosities”) measure between 0.5 and 1.5 µm; thin segments 0.15–0.4 µm. Varicose neurites account for most of the volume of the neuropile. A variant of the varicose neurites are “globular neurites”; principally similar in shape than the former, they have swellings (“globules” or “boutons”) that are more voluminous than varicosities, exceeding 1.5 µm in diameter. The globules of these central nerve fibers resemble the endplates, or “boutons,” of peripheral motor axons. The fourth class of neurites (“dendritiform neurites”) is formed by highly branched, thin (less than 0.2 µm) fibers that frequently change direction (Figure 4C, D).

Synapses are ultrastructurally defined by their characteristic presynaptic membrane specializations, consisting of an electron dense membrane thickening bordered by synaptic vesicles and the T-bar (also called synaptic ribbon), a cytoplasmic specialization involved in tethering and docking of synaptic vesicles ([44],[45]; Figure 4F, G). We observed that, independent of location within the brain, presynaptic sites are highly uniform in size, ranging from 0.15–0.3 µm, and are always found on the swellings of varicose and globular neurites (Figure 4E). These then constitute the terminal axons of the brain. In several cases, multiple presynaptic sites (individually defined by the synaptic ribbon) were confluent and formed band-like synaptic conglomerates. Postsynaptic sites, characterized by (relatively inconspicuous) membrane densities lacking T-bars or synaptic vesicles, are found almost exclusively on dendritiform neurites (Figure 4E) and (occasionally) on thin side branches of varicose neurites, implying that this class of fibers represents (terminal branches of) dendrites. Note that in insect neurons, the relatively strict distinction between dendrites and axons as two different types of neurites (a distinction that is quite typical for vertebrate brains) does not exist. Thus, the neuronal cell body emits a single neurite, which in the neuropile forms numerous branches that may be dendritic (i.e., postsynaptic) or axonal (i.e., presynaptic). Our data show that at the level of terminal branches, dendritic and axonal processes are mostly separate (pre- and postsynaptic sites rarely occur on the same preterminal process) and structurally distinct (presynaptic sites on large diameter neurites, postsynaptic sites on small diameter neurites).

The disparity in size between terminal axons and dendrites also accounts for the fact that most, if not all, synapses of the *Drosophila* brain are of the polyadic type, where a single (large) presynaptic profile contacts multiple postsynaptic sites. The pre- to postsynaptic ratio in most synapses ranges between three and five (see examples shown in Figure 4F, G).

### *Drosophila* Brain Microarchitecture: Domain-Specific Patterns of Neurite Morphology

**Figure 5:**
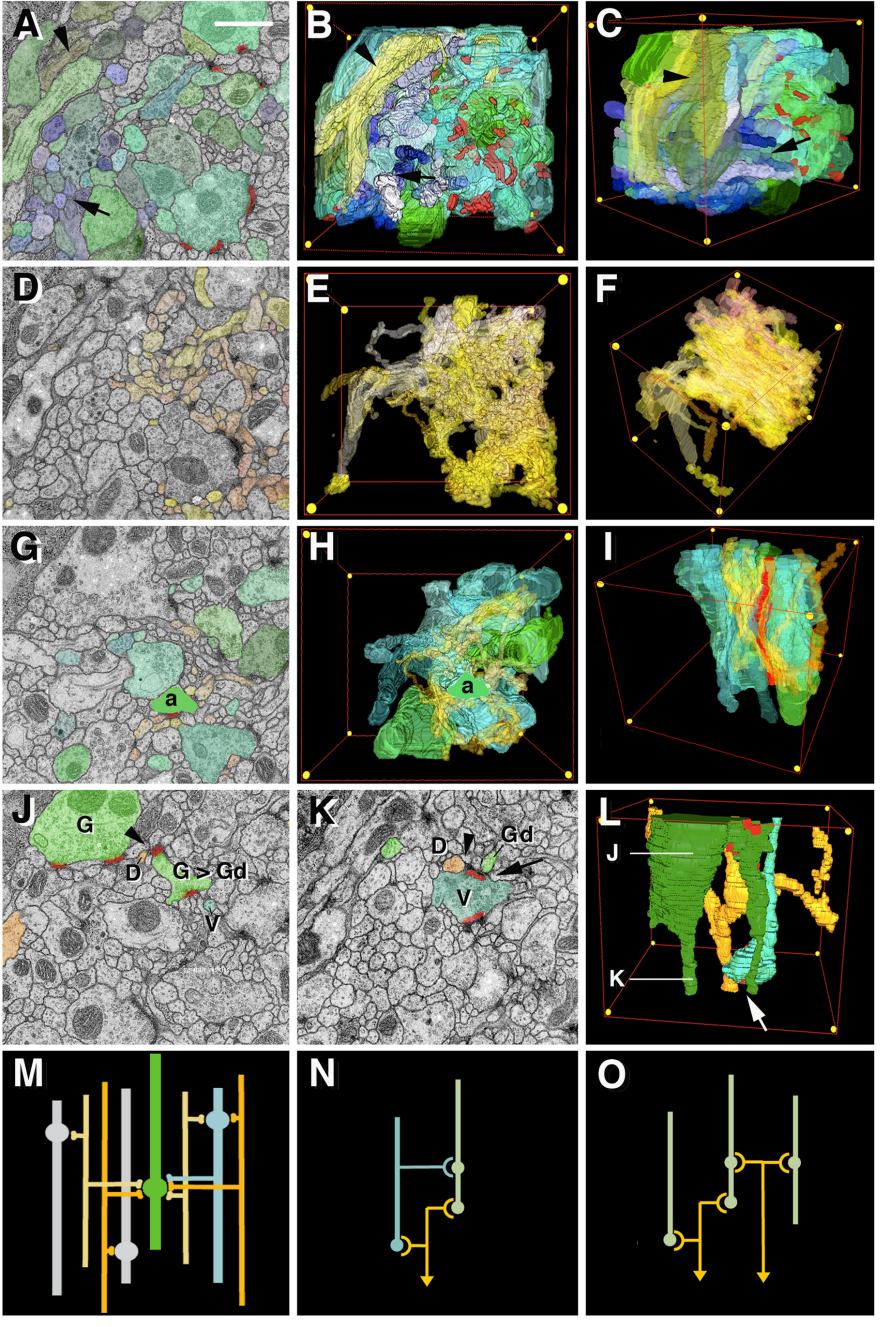
Network motifs encountered in VNC microvolume. (A–F) Structure of the VNC microvolume. (A) Representative section of VNC stack; profiles of all globular, varicose, and axiform neurites are tinted in shades of green and blue. (B, C) 3D digital model of these processes (B: frontal view; C: fronto-lateral view; red lines in these and other panels show edges of VNC microvolume; yellow dots indicate corner points). Note bundle formed by axiform neurites (arrow in A–C); another bundle of preterminal axons (light green; arrowhead) enters the microvolume from ventro-lateral. Presynaptic sites are colored red. (D) Representative section of VNC microvolume; dendritiform neurites are colored in shades of yellow and brown. (E, F) 3D digital model of all dendritiform processes in frontal view (E) and fronto-lateral view (F). (G–I) Dense overlapping regulon motif. One “primary” presynaptic neurite (“a”; bright green) contacts 12 postsynaptic dendritiform neurites (yellow-brown) at three synapses. Seven other “secondary” presynaptic elements (transparent green) are also presynaptic to these dendrites. Panel G shows representative section in which elements forming part of this motif are shaded; (H) and (I) represent 3D digital models of these elements in frontal view (H) and lateral view (I). In (I), the “primary” axonal element is colored red for better visibility. (J–L) Feed forward motif. The globular neurite (G) has thin branch (Gd) that is postsynaptic to varicose neurite segment (V; arrow in K, L). The dendritiform neurite (D) shown in orange is postsynaptic to both G (arrowhead in J) and V (arrowhead in K). Panels J and K are representative sections of VNC cube where elements of the feed forward motif are highlighted; (L) shows 3D model of motif in lateral view; levels of sections shown in (J) and (K) are indicated. (M) Schematic representation of connectivity encountered in VNC microvolume. (N, O) Schematic representation of feed forward motif and dense overlapping regulon motif, respectively. Scale bar: 1 µm (A, D, G, J, K).

Although the types of neurites depicted above are encountered in every region of the brain and ventral nerve cord, their relative numbers, directionality, branching density, and placement of synapses differ from one microvolume to the next. Two microvolumes, taken from the calyx of the mushroom body and the dorso-lateral column of the ventral nerve cord, are presented as examples (Figures 2, 5, S1).

The calyx represents the input region of the mushroom body that receives the axons of antennal projection neurons [46]. The microvolume extracted from the center of the calyx is characterized by a largely parallel array of varicose neurites carrying the majority of presynaptic sites. These neurites then most likely represent segments of the terminal axonal branches of antennal projection neurons, which project, via the antenno-protocerebral tract, to the calyx (Figure 2B). In total, the 20 µm^3^ calyx microvolume contained 33 varicose and two globular neurite segments. These neurites have an average diameter (reducing the shape of neurites to that of a smooth cylinder) of 0.38 µm. The overall “cable length” (adding together all fragments of varicose/globular neurites within the microvolume) amounted to 48 µm. There were 16 branch points, corresponding to a density of branch points of one every 3 µm of axonal cable. We find a density of about 1.8 presynaptic sites per 1 µm^3^. Dendritiform neurites form bundles of 5–10 thin fibers winding around the varicosities of preterminal axons to whom they are postsynaptic. Synapses are mostly dyadic-tetradic. Frequently, an individual dendritiform neurite participates in two or more synaptic contact made with the same axon (see also below for the VNC microvolume).

Axonal cable length, branch density, and synapse density were similar in two other brain microvolumes, one in the spur (an output region of the mushroom body; [47]) and the other in the CPLd compartment of the protocerebrum. In the spur, we counted one branch point for every 4 µm of axonal cable; in the CPLd, a branching occurred every 3.2 µm. The synapse density in the spur and CPLd was 3.3 and 1.2, respectively.

The patterning of presynaptic (varicose/globular) neurites in the dorso-lateral domain of the ventral nerve cord is characterized by a lower density of branch points (approximately one branch every 7.5 µm) and synapses per volume unit (0.8/µm^3^) when compared to the brain. Cable length and average diameter of neurites, on the other hand, equals that in the brain. All types of neurites (varicose/globular, axiform, and dendritiform) are oriented preferentially along the longitudinal axis of the ventral nerve cord (VNC; Figure 5A–F; Figure 1S). The high quality of the VNC stack (only two out of 300 sections missing) made it possible to reliably reconstruct the pattern of thin dendritiform neurites. Similar to what is seen for the brain microvolumes, these neurites have a higher branch density than varicose/globular neurites (one branch every 4.1 µm). Also, the overall dendritic cable length exceeds that of axonal elements by a factor of almost 2. Thus, the 85 µm^3^ VNC microvolume contained 247 µm of axiform/varicose/globular neurites (“axons”) and 420 µm of dendritiform neurites (“dendrites”). When following individual dendritiform processes throughout the microvolume, an interesting convergence-divergence pattern becomes apparent (Figure 4H–J). Thus, at any given level, dendritiform processes form aggregates of 5–10 processes each. However, aggregates, when following the dendritiform processes along the *z*-axis, do not translate into bundles: processes forming an aggregate at a given level stay together only for a short interval (<1 µm), after which they diverge and redistribute. This pattern reflects the fact (mentioned above) that dendritiform neurites do not follow a straight course but change direction constantly and abruptly (Figure 4C, Figure S2). By contrast, axonal processes have relatively straight trajectories: axiform neurites (long axons without synapses) form tight bundles where neighborhood relationships between neurites is maintained over manyµm (Figures 4D, H–J, 5A–C); terminal varicose/globular axons are more loosely packed but, like axiform processes, extend more or less parallel, maintaining their position relative to each other (Figures 4B, D, H–J, 5A–C).

If we take the microvolume-based measurements of cable length, branch density, and synapse density, complement them with light microscopic findings, and extrapolate to the brain as a whole, we arrive at conclusions that are both interesting and helpful for further functional/developmental analyses. The L1 brain hemisphere has a volume of approximately 20,000 µm^3^ [25]. Differentiated primary neurons whose processes make up the volume of the brain neuropile number approximately 1,500 per hemisphere [25]. The average values of presynapse density, axonal branch point density, and axonal cable length (taken from the four microvolumes) are 2/µm^3^, 0.8/µm^3^, and 2.9/µm^3^. Extrapolated to an entire brain hemisphere, this would amount to a total of 40,000 presynaptic sites, 16,000 axonal branch points, and 58,000 µm axonal cable length. For the average neuron, that means approximately 40 µm axonal length, 11 axonal branches, and 27 presynaptic sites. These estimates go along well with light microscopic data based on DiI fills (or other labelings) of L1 brain neurons. We randomly sampled L1 neuron shapes by injecting single cells with DiI and imaging neurons by fluorescence microscopy (unpublished data). It is possible to roughly estimate neurite length, and numbers of varicosities and branches from fluorescent images of labeled neurons, and obtain numbers that match closely our extrapolations from the microvolume analysis.

### Connectivity and Network Motifs in the *Drosophila* Larval Brain

An important aspect of *Drosophila* neuronal architecture is its “ultradense” neuropile architecture, for which three major structural features are responsible. First, neuronal cell bodies, in terms of volume a considerable part of the brain, are located outside of the neuropile and therefore do not form part of circuits (i.e., do not carry synapses). Second, dendritiform processes are extremely thin and highly branched. Third, synapses are polyadic, with each presynaptic site attaching to an average of four postsynaptic sites. As a result of these factors, neurites within a microvolume are highly interconnected, and one can detect in high numbers certain types of network motifs [48],[49] that may determine the function of the corresponding neuropile microvolume. We will in the following summarize our first data concerning connectivity and network motifs for the microvolume generated for the dorso-lateral VNC.

From the 85 µm^3^ VNC microvolume we reconstructed 170 elements over 1 µm length (Figures S1, S2). Of these, 39 were varicose neurites; one was globular, 25 axiform, and 105 dendritiform. We counted 68 presynaptic sites, all concentrated on the large diameter segments of the globular neurite and of 24 varicose neurites. Only a single presynaptic site was found on an axiform process. Among the 24 neurites with synapses, the synapse number per neurite ranged between one and nine (average 2.7). The dendritiform processes formed 256 postsynaptic sites, yielding an average ratio of four postsynaptic sites to every one presynaptic site. In turn, each dendritiform process contacted an average of 2.1 presynaptic neurites.

**Figure 6:**
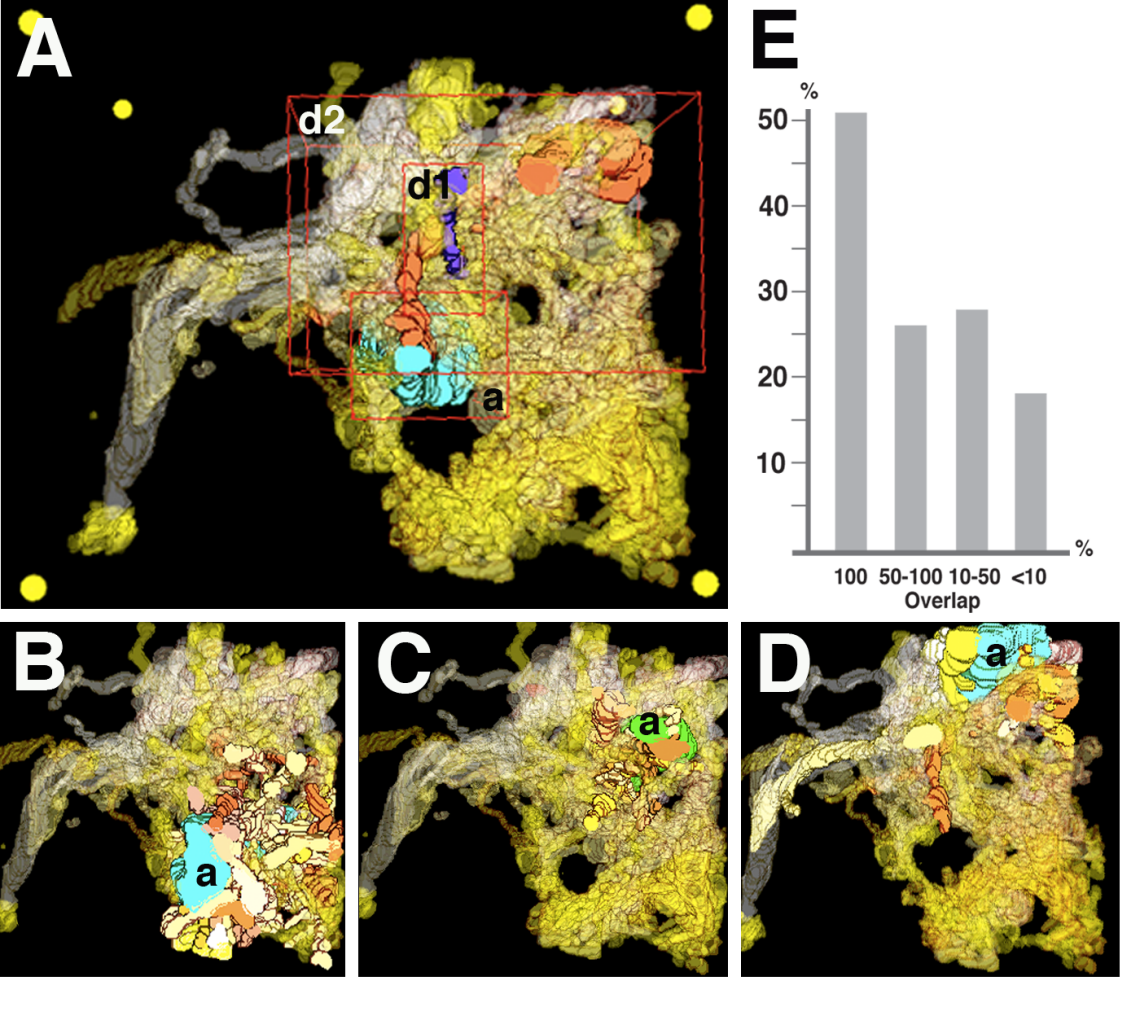
Correlation between spatial overlap of neurite segments and probability of synaptic contacts in VNC microvolume. Panels A–D show 3D digital models of elements of VNC microvolume (frontal view). All dendritiform neurites are rendered in transparent shades of yellow-brown. In panel A, one axiform neurite (a) and two dendritiform neurites (d1, d2) are rendered in solid colors. Red frames delineate boundaries of envelope surrounding the three profiles. In this example, envelope of d1 and a overlap slightly (less than 10% of volume of envelope of a is shared with envelope of d1); envelope of d2 and a overlap strongly (greater than 50% of volume of envelope of a is shared with envelope of d2). In each one of panels B–D, one varicose neurite (a) is rendered in solid green or blue; the postsynaptic dendritiform neurites that actually form synaptic contacts with the varicose neurite are rendered in solid colors (yellow-brown). Note tight clustering of denritiform neurites around the corresponding varicose element. (E) Histogram depicting correlation between overlap of pre- and postsynaptic elements (*x*-axis) and frequency of synaptic contacts (*y*-axis). Counted for the VNC microvolume; overall number of presynaptic elements over 1 µm length: 40; postsynaptic elements: 105. For example, only 17% of the pre- and postsynaptic elements whose envelopes overlap less than 10% actually form synaptic contacts.

Contacts between neurites of the VNC microvolume are highly concentrated along the longitudinal (*z*) axis. In other words, postsynaptic partners of a given, longitudinally oriented presynaptic process are clustered closely around this process (Figures 5M, 6). To quantify the correlation between the likelihood of forming a synaptic contact and distance between two processes, we visualized the envelopes that included a given axon and the dendritiform neurites (Figure 6A) and then estimated in a pairwise fashion the amount of overlap between axonal and dendritic envelope (complete overlap, more than 50%, 10%–50%, less than 10%). For each class we determined the frequency at which a synaptic contact is made (Figure 6E). This frequency is a function of overlap (which in turn reflects distance): if the overlap exceeds 50%, more than half of the dendritic processes form synapses with the corresponding axon; this drops to about a quarter at an overlap between 50% and 10% and to 17% at less than 10% overlap (Figure 6B).

Most of the interconnected pre- and postsynaptic elements of the VNC microvolume engaged in what is called a “dense overlapping regulon motif” [49], defined as a network where a given input element (axon) diverges onto multiple targets, and at the same time, each target element (dendrite) receives input from multiple presynaptic elements (Figure 5M, O). In the example shown in Figure 5G–I, one varicose neurite possesses three presynaptic sites on two varicosities. Contacting these sites are 12 postsynaptic dendritiform processes. Each dendrite in turn receives input from up to five presynaptic partners. Most of the 24 axonal processes within the VNC microvolume formed part of such a dense overlapping regulon network.

A second type of network, encountered much less frequently, is the feed forward motif. Here, a neurite, “A,” receives input from a second neurite, “B,” and both “A” and “B” provide output to a common target, “C” (Figure 5M, N). Thus, feed forward motifs involve instances of neurites that serve as presynaptic partners to some and postsynaptic partners to other fibers. In the VNC microvolume, we encountered five cases where varicose neurites, which are mostly presynaptic, also formed a postsynaptic site. In all cases, this postsynaptic site was located not on the varicosity itself, but the thin segment or a finger-like side branch of the neurite (Figure 5J–L). In two cases, axiform neurites had postsynaptic sites. Interestingly, fibers that, based on the presence of characteristic dense core vesicles, belong to peptidergic neurons all form part of axo-axonal contacts. In three cases, they served as presynaptic partners, and in two cases as postsynaptic partners.

## Discussion

The approach towards the analysis of microcircuitry introduced here makes it possible to analyze and compare ultrastructural features of the neuropile across the entire brains. Our data show that there may exist a number of generic properties of neuropiles, which in part have already been observed in previous studies of insect brain neuropile. In all parts of the central nervous system, we could distinguish between four different types of neurite profiles, globular/varicose, axiform, and dendritiform. The swellings of globular/varicose neurites are terminal axonal branches, carrying presynaptic sites; these presynapses are contacted by multiple, small-diameter dendritiform processes. Out of the several hundred synapses observed, less than a percent did not conform to this polyadic type. It is noteworthy that representative sections of late larval and adult central brain neuropile (VH, unpublished), as well as TEM sections of neuropile documented in the literature [13],[14],[50]–[54], showed that the diameters of the large majority of presynaptic and postsynaptic profiles, and the structure of synapses, are very similar to what we see in the early larval brain of *Drosophila*.

It is a widely reported finding that the dendrites and axons of invertebrate neurons are not spatially separated as in most vertebrate neurons but occur intermingled. Thus, a typical insect neuron may produce one or a few major stem branches that give off higher order branches with either dendritic or axonal properties. What emerges from the literature is that in most cases, the arbor of a given neuron can be subdivided into territories that are predominantly (but not exclusively) presynaptic or postsynaptic. For example, synaptic sites of antennal projection neurons are predominantly postsynaptic in the antennal lobe (close to the cell bodies of these neurons), and presynaptic in the calyx (where they form the massive “microglomeruli  =  globular endings; [13],[55]). But even the proximal neurites in the antennal lobe were found to carry presynaptic sites [53]. Significantly, these were found on the proximal, large diameter neurites; as these neurites branched into higher order, thinner terminal fibers, these were all postsynaptic. This report is in line with our finding that almost without exception, postsynaptic profiles were of very small diameter, and presynaptic profiles of large diameters. It is reasonable to assume that the generic neurite properties that we establish here for different neuropile compartments of the larval central brain and ventral nerve cord may find close parallels in other insect brains as well. The computer-aided recording and reconstruction of serial TEM sections applied in this work makes it feasible to generate data sets in other species in a reasonable amount of time, which gives good reason to anticipate that quantitative ultrastructural data concerning neuropile architecture will be available soon for a variety of different brains.

### Neuropile Architecture in Mammalian Cortex and Fly Brain: A First Comparison

Statistical analyses of light microscopic preparations (e.g., Golgi stained preparations) and representative EM sections of mammalian brains yielded estimates for parameters like synapse density, cable length, and branch point density (e.g., [10]) that can be compared to the data we present here for the fly larval brain. Surprisingly, when considering the enormous difference in overall size of individual neurons and the brain as a whole, a number of parameters are very similar. For example, the average diameter of axon shafts and synapse bearing varicosities, as well as presynaptic sites themselves, is almost the same in *Drosophila* brain and mammalian cortex. As a result, the overall axonal cable length per neuropile volume is also very similar. One mm^3^ of mouse cortex contains an estimated 4,100 m of axon; translating this number to a smaller cube of 10 µm length, more appropriate when dealing with miniature brains, would yield 4.1 mm of axon. In the microvolumes analyzed for the larval brain, axonal cable length ranged from 1.5 mm to 4.0 mm in a cube of 10 µm length. A conspicuous difference between the mammalian cortex and fly brain lies in the size of dendrites. In mammals, dendrite shafts have an average diameter of close to 1 micrometer [10], and many dendrites are considerably thicker and less branched. As a result, dendritic cable length per volume unit appears to be much lower than axonal cable length in mammalian brain (0.5 versus 4.1 mm in a 10×10×10 µm cube), whereas the opposite is true in fly brain. Thus, when extrapolated to a 10×10×10 µm volume, the overall length of varicose/globular neurites totaled 2.9 mm; that of dendritiform neurites was 5.4 mm.

Another distinguishing characteristic of the *Drosophila* brain appears to be the higher density of branch points, in particular for dendrites. Statistical analyses in mammalian cortex yielded average distances of 10 µm and higher between branch points [10]. Measured here for *Drosophila*, terminal axons had average branch point intervals of 4 µm (mushroom body calyx), 2.8 µm (mushroom body, spur), or 7.5 µm (VNC), respectively. The density of branch points in dendrites appears to be even higher. Thus, for the VNC cube where thin dendrites could be reliably reconstructed, we measured an average interbranch point distance of 4.9 µm.

In light of the higher branch point density, one might also expect a higher number of synaptic contacts per volume unit in *Drosophila*, compared to the mammalian brain. In mammalian cortex, synaptic density has been estimated at 0.72/µm^3^ [10]. Synapses are predominantly of the monadic type, where one presynaptic site contacts a single postsynaptic site. By contrast, synapses are polyadic in the *Drosophila* nervous system. For each presynaptic site, one observes multiple postsynaptic sites. In terms of total number of synaptic contacts (where one would count a tetrad with three postsynaptic partners as three contacts), the *Drosophila* brain does have a much higher synapse density than the mammalian brain; when only counting presynaptic sites, numbers are comparable. Thus, in the material analyzed for this study, presynapse density falls within a range from 0.8/µm^3^ (VNC) to about 4/µm^3^ (input region of mushroom body). We expect that for mammalian brain, additional direct measurements will yield values of synapse density that may considerably vary between different neuropile compartments.

The higher density of branches and synaptic contacts in the *Drosophila* brain, compared to mammalian brain, is correlated with a larger degree of “connectedness” between neurites. As shown in this study, the large majority of neurites contained within a microvolume of less than 100 µm^3^ are engaged in networks of the type of dense overlapping regulon or feed forward motifs, which simply reflects the fact that in average, terminal axons and dendrites in such a small volume have multiple branches that engage in synaptic contacts. This is not the case in mammalian cortex. When considering the low average branch point density (one every 10 µm; see above), it is unlikely that many neurite segments included within a 100 µm^3^ volume will have a branch, and thereby more than one contact. This estimate is confirmed in a recent microvolume reconstruction of rat hippocampus [18],[56]. Here, the only type of connectivity observed consists in the convergence of multiple axonal segments onto isolated dendritic segments. However, very few of the (unbranched) axonal segments give synaptic input to more than one (unbranched) dendritic segment. As a result, network motifs like the dense overlapping regulon motif or feed forward motif are not observed within microvolumes of 5 µm diameter. In other words, the volumes of mammalian brain that “contain” microcircuits are considerably larger than in *Drosophila*: connectivity occupies more space. The number and size of vertebrate neurons are typically much larger than in insects. Other fundamental architectural features of vertebrate brains are the inclusion of neuronal somata into the neuropile (somata carry many postsynaptic sites), the large diameter of dendrites, and concomitantly, the absence of polyadic synapses. One may speculate that one of the prime driving forces of the evolution of vertebrate brain architecture was the increasing number of neurons. Consecutively, the average vertebrate neuron also had to grow individually in axonal and dendritic length, in order to accommodate the higher number of synapses needed to connect a given neuron to a certain fraction of the (increasing) pool of neurons. Finally, as a result of the increase in cell number, cable length, and synapse number, branches of dendrites and axons became spaced further apart. Typical “microcircuits” in the mammalian brain, formed by multiple interconnected elements providing for stimulus convergence and divergence and inhibitory/excitatory feed forward and feed back loops, will occupy volumes of (300–1,000 µm)^3^ diameter [7],[57],[58]. In the *Drosophila* brain, these volumes would be two orders of magnitude smaller, which constitutes a significant advantage when reconstructing circuitry from densely segmented serial EM sections.

### Digital Serial EM Analysis as a Tool for the Genetic and Cell Biological Analysis of Neuronal Development

The dense segmentation of serially sectioned neuropile plays a pivotal role for reconstructing microcircuitry. However, the ability to reconstruct ultrastructural features of neurites in all parts of the neuropile of a small brain, such as the *Drosophila* larval brain, will prove to be of great value for in vivo cell biological and genetic studies of neuronal development as well. The complex shape and connectivity of a neuron is a reflection of an intracellular molecular machinery that places membrane proteins (e.g., adhesion molecules, receptors, channels) and cytoskeletal proteins into the right position, such that pre- and postsynaptic sites, branch points, and specific connections with targets are formed in the right pattern. The analysis of these developmental phenomena is in its infancy. We know of many proteins that are differentially expressed in specific membrane domains [59],[60] and that the directional protein transport in axons and dendrites is controlled by different mechanisms. However, the exact mechanisms of targeted protein expression are unknown. Models such as *Drosophila* offer the opportunity of a genetic approach, that is, study the phenotype following genetic manipulation. This approach has been very successful to elucidate the development of several types of (peripheral) sensory neurons and motor neurons [61]–[63]. However, up until now, virtually no mutant phenotypes have been established for central brain neurons or circuits on the ultrastructural level, simply because the basic parameters of wild type neuronal ultrastructure were not available, and the necessary technology, serial EM, was too time-consuming. With the computer-aided reconstruction of EM stacks, such an analysis is now within reach. Thus, once parameters of neurite size, branching, and connectivity have been established for a number of compartments, it will be possible to generate stacks of EM sections that contain specific brain compartments (such as the calyx or lobes of the mushroom body) of genetically manipulated animals, and carry out detailed, quantitative comparisons with the wild-type. Such stacks (for the early larval brain) would contain as little as 100 contiguous sections; their preparation, image capturing, and analysis (when following the microvolume approach) may require only a matter of weeks. It is therefore realistic to generate EM stacks numerous specimens' brains of larvae carrying specific mutations, to then establish changes in basic neurite parameters, such as diameters, branch and synapse density, and synapse architecture.

### Approaches to the Dense Reconstruction of Neuropile

Serial TEM is the classical approach for the reconstruction of microcircuitry. In the days before digital photography and computer-assisted image processing, this approach was extremely labor intensive and was therefore utilized mostly for small parts of individual neurons (“sparse segmentation”). The only exception was the reconstruction of the *C. elegans* central nervous system [64],[65]. We predict that given the speed of imaging that is now possible (and will certainly further increase), serial EM will experience a renaissance as an approach for the reconstruction of microcircuitry.

Two recent technological developments have reduced the difficulty of large-scale serial section electron microscopy and improved its reliability. Serial block-face scanning electron microscopy [66] has proven useful in imaging relatively large volumes of neural tissue at an isotropic resolution of about 20 nm/pixel. The smaller dimensions of *Drosophila* neural tissue components, compared to the vertebrate equivalents, require a resolution of at least 8 nm/pixel (ideally 4 nm/pixel) for the reconstruction of small terminal dendrites, which is necessary for the conclusive elucidation of synaptic partners. The second novel technique, focused ion beam (FIB) milling combined with block-face scanning electron microscopy [67], delivers up to 5 nm/pixel resolution. While FIB delivers images with the necessary resolution for the reconstruction of *Drosophila* neuropile, its imaging field of view is currently limited in practice to a window of 20×20 microns (Graham Knott, personal communication), which does not enclose a transverse section of the entire nerve cord neuropile of *Drosophila* larva. Traditional serial section transmission electron microscopy, as employed in this article, delivers the required high imaging resolution on the plane (4 nm/pixel or better) but reduced resolution in the *z*-axis (50 nm, the thickness of the section). However, our reconstructions indicate that, given sufficient resolution in the XY plane, the 50 nm/pixel resolution of the *z*-axis does not prevent the full reconstruction of even the smallest terminal dendrites, measuring only about 60 nm in diameter. We anticipate that for the immediate future, serial TEM, BF/SEM, and FIB/SEM will coexist as more or less equally valuable techniques for the electron microscopic reconstruction of microcircuitry.

The generation of large EM image data sets has made imperative the development of novel specialized software for its processing, visualization, and analysis [15]. The sheer size of the data sets has prompted the development of novel algorithms for the automatic segmentation and reconstruction of neural arbors [18]. Thus, given the current conditions, to manually segment an entire L1 Drosophila brain would take one person in the order of 50 years, which means that the development of tools for automatic segmentation is of high priority. It should be noted that the 50-year projection does not take into account the fact that neuropile ultrastructure, to some extent, is most likely modular. As a result, the network diagram extracted from a given microvolume can be extrapolated to neighboring volumes, as long as they fall within the same compartment. In other words, the hope and anticipation (in particular in regard to “big brains” of vertebrates) is that one does not dense-segment every single “voxel” of a given compartment but focus on a certain set of samples that are (manually or automatically) densely segmented; then, using algorithms that need to take into account data from many microvolumes, circuitry in the samples can be extrapolated to a compartment as a whole.

Until recently, two software packages were primarily used for visualization and analysis of TEM serial sections: IMOD [17] and Reconstruct [16]. IMOD is the current gold standard in EM software for image composition and processing. Reconstruct provides an efficient GUI for manual and semiautomatic image stitching and is particularly noted for its manual image segmentation toolkit. Neither of these programs handles data sets that are considerably larger than RAM and have limited support for large-scale image registration with nonlinear transformations. The ir-tools and associated visualization applications [15], while providing effective image registration and a comprehensive image analysis toolkit, depend on RAM and lack ease of customization for highly specialized tasks. We have found that TrakEM2, while not yet fully independent of RAM, provides the means to register hundreds of thousands of images, overcoming numerous limitations imposed by computer hardware. TrakEM2 eases the concatenation of image transformations, including polynomial models for lens deformation correction [32]. In contrast to the ir-tools, TrakEM2 operates on image tiles that correspond to the original acquired images and not on stitched large images. Precomputed image pyramids for each tile enable enhanced performance. We highlight the robustness of the image registration library associated with TrakEM2, and the ability to manually correct errors when these inevitably occur, an ability facilitated by the tile-oriented approach. Finally, TrakEM2 is a (large) component of Fiji [68], an ImageJ-based image processing environment in active development and with thousands of image analysis plugins readily available. We believe that TrakEM2 represents a useful addition to existing software packages that can handle special tasks, in particular the segmentation and subsequent analysis of large data sets with high numbers of individual elements. In summary, TrakEM2 improves over existing software packages in being less dependent on scarce computer resources and by bundling numerous image segmentation and analysis tools within a unique graphical interface. TrakEM2 acknowledges that any automatic procedure (such as image registration and image segmentation) will eventually fail partially or fully and will require manual correction by a human operator. The combination of both manual and automatic procedures for neuronal reconstruction makes TrakEM2 a practical application for the reconstruction of large volumes of brain neuropiles.

## Supporting Information

Figure S1 **Digital 3D models of all varicose/globular and axiform elements over 1 mm length segmented from VNC microvolume.** All panels in lateral view; anterior to the left, dorsal up. Rows 1–8 show globular/varicose elements; red dots represent presynaptic sites. Rows 9–13 show axiform elements. Scale bar: 1 µm.
(6.70 MB TIF)

Figure S2 **Digital 3D models of all dendritiform elements over 1 mm length segmented from VNC microvolume.** All panels in lateral view; anterior to the left, dorsal up. Vertical elements of last row [15] formed a bundle of relatively large diameter fibers that grazed lateral surface of VNC microvolume; the basis for classifying them as dendritiform was that they possessed short segments or branches approaching the neuropile. Scale bar: 1 µm.
(9.61 MB TIF)

## Abbreviations

BF/SEM: block face scanning electron microscopy
CPLd: centro-posterior-lateral compartment, dorsal domain
dePF: descending protocerebral fascicle
EM: electron microscopy
FIB/SEM: focused ion beam scanning electron microscopy
GFP: green fluorescent protein
GUI: graphical user interface
L1: Drosophila first larval instar
LM: light microscopy
MEF: medial equatorial fascicle
MRI: magnetic resonance imaging
PAT: primary axon tract
PB: phosphate buffer
PBS: phosphate buffered saline
PBT: phosphate buffered saline with Triton X-100
PDF: pigment dispersing factor
PET: positron emission tomography
SIFT: scale-invariant-feature-transform
TEM: transmission electron microscopy
VNC: ventral nerve cord
