# Supplementary figures and images for: An Integrated Micro- and Macroarchitectural Analysis of the Drosophila Brain by Computer-Assisted Serial Section Electron Microscopy
Source: PLoS Biol. 2010 Oct 5;8(10):e1000502. doi: 10.1371/journal.pbio.1000502 (PMC2950124; doi:10.1371/journal.pbio.1000502)

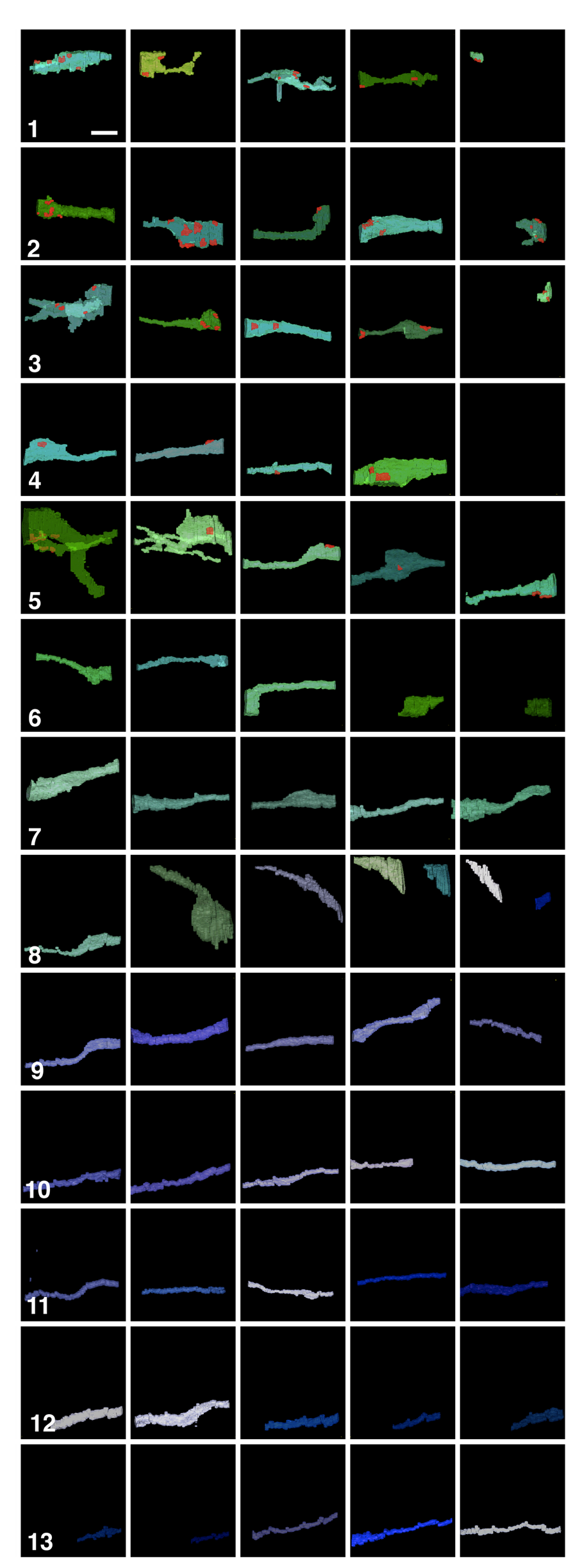

Supplement: Figure S1 — Digital 3D models of all varicose/globular and axiform elements over 1 mm length segmented from VNC microvolume. All panels in lateral view; anterior to the left, dorsal up. Rows 1–8 show globular/varicose elements; red dots represent presynaptic sites. Rows 9–13 show axiform elements. Scale bar: 1 µm. (6.70 MB TIF) [file pbio.1000502.s001.tif]

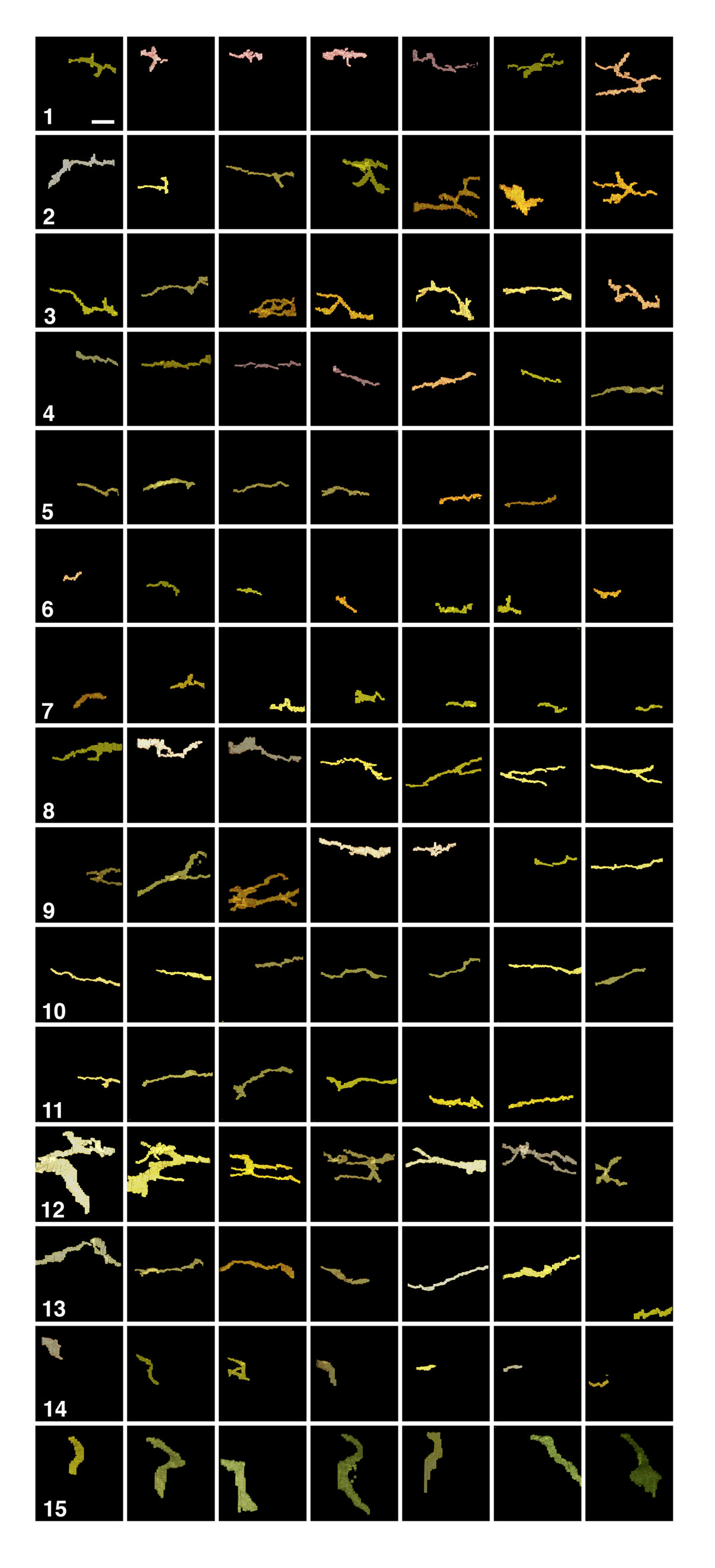

Supplement: Figure S2 — Digital 3D models of all dendritiform elements over 1 mm length segmented from VNC microvolume. All panels in lateral view; anterior to the left, dorsal up. Vertical elements of last row [15] formed a bundle of relatively large diameter fibers that grazed lateral surface of VNC microvolume; the basis for classifying them as dendritiform was that they possessed short segments or branches approaching the neuropile. Scale bar: 1 µm. (9.61 MB TIF) [file pbio.1000502.s002.tif]
